# Toxicity of silver nanoparticle in rat ear and BALB/c 3T3 cell line

**DOI:** 10.1186/s12951-014-0052-6

**Published:** 2014-12-03

**Authors:** Jing Zou, Hao Feng, Marika Mannerström, Tuula Heinonen, Ilmari Pyykkö

**Affiliations:** Hearing and Balance Research Unit, Field of Oto-laryngology, School of Medicine, University of Tampere, Tampere, Finland; The Finnish Centre for Alternative Methods, School of Medicine, University of Tampere, Tampere, Finland; Department of Otolaryngology-Head and Neck Surgery, Center for Otolaryngology-Head & Neck Surgery of Chinese PLA, Changhai Hospital, Second Military Medical University, Shanghai, China

**Keywords:** Animal model, Biological barrier, Ear, Imaging, Nanomaterial

## Abstract

**Background:**

Silver nanoparticles (AgNPs) displayed strong activities in anti-bacterial, anti-viral, and anti-fungal studies and was reportedly efficient in treating otitis media .The potential impact of AgNPs on the inner ear was missing.

**Objective:**

Attempted to evaluate the potential toxicity of AgNPs in the inner ear, middle ear, and external ear canal after transtympanic injection in rats.

**Results:**

In in vitro studies, the IC_50_ for AgNPs in neutral red uptake assay was lower than that in NAD(P)H-dependent cellular oxidoreductase enzyme assay (WST-1) and higher than that in total cellular ATP and nuclear membrane integrity (propidium iodide) assessments. In in vivo experiments, magnetic resonance imaging (MRI) showed that significant changes in the permeability of biological barriers occurred in the middle ear mucosa, the skin of the external ear canal, and the inner ear at 5 h post-transtympanic injection of AgNPs at concentrations ranging from 20 μg/ml to 4000 μg/ml. The alterations in permeability showed a dosage-response relationship, and were reversible. The auditory brainstem response showed that 4000 μg/ml AgNPs induced hearing loss with partial recovery at 7 d, whereas 20 μg/ml caused reversible hearing loss. The functional change in auditory system was in line with the histology results. In general, the BALB/c 3T3 cell line is more than 1000 times more sensitive than the in vivo studies. Impairment of the mitochondrial function was indicated to be the mechanism of toxicity of AgNPs.

**Conclusion:**

These results suggest that AgNPs caused significant, dose-dependent changes in the permeability of biological barriers in the middle ear mucosa, the skin of the external ear canal, and the inner ear. In general, the BALB/c 3T3 cell line is more than 1000 times more sensitive than the in vivo studies. The rat ear model might be expended to other engineered nanomaterials in nanotoxicology study.

**Electronic supplementary material:**

The online version of this article (doi:10.1186/s12951-014-0052-6) contains supplementary material, which is available to authorized users.

## Background

Chronic otitis media, characterized by recurrent infections causing pain and purulent otorrhea, is still a significant public health problem affecting 0.5–30% of any given population in developing and developed countries. However, antibiotic is not always efficient because of the appearance of multidrug resistant strains of bacteria. Formation of biofilm was recently reported in the middle ear of patients with chronic otitis media all over the world [1-4]. Silver nanoparticles (AgNPs) displayed strong activities in anti-bacterial, anti-viral, and anti-fungal studies attributed to the mechanisms of inhibiting the formation of biofilm and destroying viral structures and boosting innate immune response among others [5-9]. The medical applications of AgNPs include surgical fields, such as urology, dentistry, general surgery and orthopedics, and wound dressing to take advantage of good antibacterial activity [10]. Therefore, AgNPs will potentially be selected as an alternative strategy to treat diseases in the ear by combating biofilm formation and any potential multidrug resistant strains of bacteria that is big challenge for conventional antibiotics. A clinical study on treatment of relapses of chronic suppurative otitis media showed that a preparation containing silver nanoparticles eliminated clinical symptoms and positive dynamics of the objective signs of the disease, such as reduction or termination of pathological exudation and stimulation of the epidermization processes, which was stable during the observation time of 6 months [11]. However, before formal application in the clinic practice, sophisticated toxicological study on AgNPs in the inner ear to evaluate any potential risk of the new agent is necessary.

In vivo rodent studies have shown that AgNPs induce liver and neural toxicity after intravenous injection [12]. The neural toxicity in the brain is suspected to be the result of the passage of AgNPs across and breaking down the blood–brain barrier [13]. Biological barriers are defined as a membrane, tissue, or mechanisms that selectively transport certain substances into the tissue and block others. Previous work showed that a rat’s inner ear has also a sophisticated barrier system isolating different compartments as well as the engineered nanomaterials distributed in the inner ear after middle ear administration [14-17]. This membranous barrier system in the inner ear is similar to that in the brain and the functional changes in the barriers resulting from hazardous exposure can be evaluated using MRI with high accuracy [18,19]. The auditory function alteration caused by toxic substances can also be measured accurately, which is otherwise inconvenient in the brain or cranial nerves [15,20]. The blood-endolymph and blood-perilymph barriers control the interaction between the inner ear and blood. The blood-perilymph barrier is permeable to certain small molecules and is similar to that of cerebrospinal fluid, with minor variations [21-23]. The blood-endolymph barrier is as tight as the blood–brain barrier and does not allow the MRI contrast agent, gadolinium chelate, to pass through under physiological conditions [24,25]. AgNPs may pass through and impair these barriers in the inner ear. In addition, the skin in the external ear canal and mucosa in the middle ear cavity are also exposed to AgNPs when the agent is delivered to the external ear canal to treat otitis media. Transtympanic injection of AgNPs in rats can mimic the clinical application and the animal suffering is minor. This multifunctional rat ear model can also be utilized for evaluating potential toxicity of other types of engineered nanomaterials with a focus on the impacts on the biological barriers in the skin (external ear canal), mucosa (middle ear cavity), nerve system (inner ear).

In the work presented here, *in vitro* study was performed in the BALB/c 3T3 cell line that were exposed to AgNPs for 24 h, a longer exposure time than the literature report in order ensure adequate toxicity [26]. Using the neutral red uptake (NRU) assay which is an *in vitro* evaluation of acute mammalian toxicity accepted by Organization for Economic Cooperation and Development (OECD GD 129, 2010) [27] (http://www.alttox.org/ttrc/validation-ra/validated-ra-methods.html). The NRU study was further compared with three other cytotoxicity assays with different end points: NAD(P)H-dependent cellular oxidoreductase enzyme assay (WST-1) for evaluating the mitochondrial function (which produces a water-soluble formazan, reacts with the mitochondrial respiratory Complex II, and is more stable than conventional MTT assay) [28], the total cellular ATP measurement as a general indicator of mitochondrial activity, and the propidium iodide staining for assessing the nuclear membrane integrity that are alternative methods. Cytotoxicity of AgNPs was compared with AgCl (in the case of Ag^+^ release, AgCl is the major product in the body), as well as AgNO_3_ as a material control that was reported in the literature [29]. *In vivo* experiments were carried out in rats after transtympanic injection of either AgNPs, or solution of AgNO_3_ or AgCl. Functional changes in the blood-inner ear barriers, as well as in the capillary barriers in the skin of the external ear canal and mucosa of the middle ear cavity, were evaluated using gadolinium-enhanced magnetic resonance imaging (MRI). Auditory function was monitored by the auditory brainstem response (ABR) measurement. The potential cell death of different cellular populations in the cochlea was analyzed.

## Results

### Comparison of AgNP toxicity in BALB/c 3T3 cells between NRU and other end points

**Figure 1:**
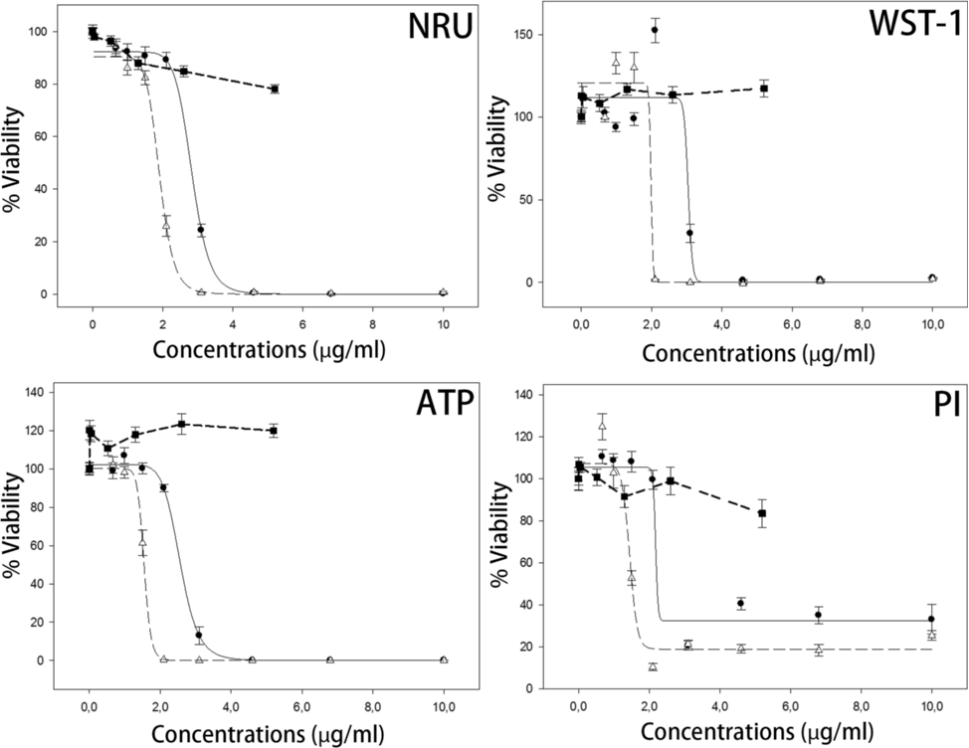
**Cytotoxicity of AgNPs, AgNO**_**3**_ **, and AgCl in BALB/c 3T3 cells shown by NRU, WST-1, total cellular ATP, and nuclear membrane integrity assessments.** −• − AgNPs, −-Δ-- AgNO_3_ solution, −-■ -- AgCl solution.

The IC_50_ for AgNPs was 2.8 μg/ml in NRU assay, which was lower than that in WST-1 and higher than that in total cellular ATP and nuclear membrane integrity (propidium iodide) assessments (Figure 1, Table 1). There was a significant (P < 0.001) ~50% increase in mitochondrial activity of BALB/c 3T3 cells at AgNP concentration 2.1 μg/ml (shown by WST-1). Thereafter the viability dropped steeply. Other cytotoxicity assays showed no such increase in viability compared to control value (untreated cells). The IC_50_ for AgNO_3_ was lower than that for the AgNPs in all the measurement methods. However, AgCl up to saturated concentration was insignificantly toxic.

**Table 1:** **IC**_**50**_ **of AgNPs and AgNO**_**3**_ **evaluated in BALB/c 3T3 cells using NRU, WST-1, ATP, and PI methods**

**Assay**	**IC**_**50**_ **(μg/ml) and coefficient of determination (R** ^**2**^ **)**			
	**AgNPs**		**AgNO**_**3**_	
	**IC**_**50**_	**R** ^**2**^	**IC**_**50**_	**R** ^**2**^
**NRU**	2.8	0.97	1.9	0.97
**WST-1**	3.0	0.86	2.0	0.93
**ATP**	2.6	0.96	1.5	0.95
**PI**	2.2	0.80	1.4	0.78

ATP, total ATP measurement; NRU, neutral red uptake assay; PI, propidium iodide penetration assay; WST-1, NAD(P)H-dependent cellular oxidoreductase enzyme assay.

### Permeability change of the biological barriers in rat ear after AgNP exposure

**Figure 2:**
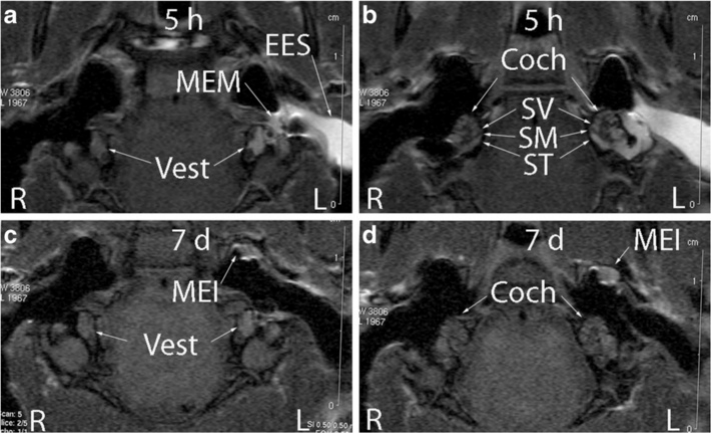
**Gd-contrasted MRI showed that dynamic changes in the permeability of the biological barriers occurred in the middle ear mucosa, skin of the external ear canal, and the inner ear after exposure to 400 μg/ml AgNPs.** Figures **a** and **b** are measured 5 h after exposure and figures **c** and **d** 7 d after exposure. Quantification data was shown in Figure 3. Coch: cochlea; MEI: middle ear infiltration; MEM: middle ear mucosa; SM: scala media; ST: scala tympani; SV: scala vestibuli; Vest: vestibulum.

**Figure 3:**
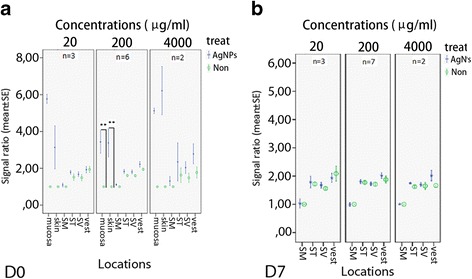
**Quantifications on AgNP induced permeability changes in the biological barriers in the skin, mucosa, and inner ear shown by Gd-DOTA-enhanced MRI. a**. Results on d 0. **b**. Results on d 7. *p < 0.05; **p < 0.01(independent t-test). SM: scala media; ST: scala tympani; SV: scala vestibule; vest: vestibule.

Significant changes in the permeability of the biological barriers occurred in the middle ear mucosa, skin of the external ear canal, and the inner ear at 5 h post-transtympanic injection at concentrations ranging from 20 μg/ml to 4000 μg/ml. The induced permeability alteration showed a dosage-response relationship and recovered to base line in barriers in the middle ear mucosa, external ear canal skin, and the inner ear except vestibule, that only partially recovered at 7 d post-exposure to concentrations lower than 4000 μg/ml of Ag NPs (Figures 2 and 3).

### The impact of AgNP exposure on the auditory function of rats

**Figure 4:**
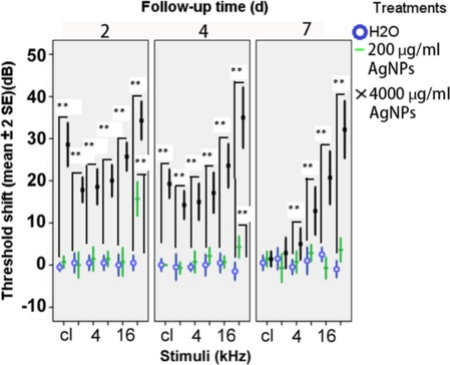
**The threshold shifts in the rats caused by the transtympanic injection of the AgNPs are shown by auditory brainstem response (ABR) measurements.** 40 μl of either AgNPs at different concentrations or water were delivered into the middle ear via transtympanic injection. The ABR were measured before and after injection at defined time points. The threshold shift = the threshold post injection-threshold pre-injection. cl: click. **p < 0.01 (independent sample t-test).

ABR is an auditory evoked potential extracted from ongoing electrical activity in the brain and recorded with subcutaneous platinum needle electrodes placed in the scalp. The resulting recording is a series of vertex positive waves of which I through V are evaluated. The wave II is the most evident and stable response in rats and utilized to identify the threshold which is the minimum visible and repeatable response. The hearing loss level was presented as threshold shift in the ABR measurement, which is that the larger the threshold shift the more severe the hearing loss. The increase in this parameter indicates the degree of hearing loss that is expressed by decibel (dB). Figure 4 showed the induced hearing loss in rats. At 2 d post-transtympanic injection, the AgNPs at a concentration of 4000 μg/ml caused significant threshold shifts of 29 dB upon click stimuli and from 18 dB to 32 dB with tone burst stimuli at frequencies of 2 kHz, 4 kHz, 8 kHz, 16 kHz, and 32 kHz (p < 0.01, independent sample t-test); however, AgNPs at a concentration of 200 μg/ml caused only a significant threshold shift of 16 dB at 32 kHz (p < 0.01, independent sample t-test). On 4 d post-transtympanic injection, AgNPs at a concentration of 4000 μg/ml caused significant threshold shifts of 19 dB upon click stimuli and from 14 dB to 35 dB on tone burst stimuli at frequencies of 2 kHz, 4 kHz, 8 kHz, 16 kHz, and 32 kHz (p < 0.01, independent sample t-test). The threshold shift induced by AgNPs at a concentration of 200 μg/ml reduced to 4 dB at 32 kHz but remained significant (p < 0.01, independent sample t-test). At 7 d post-transtympanic injection, AgNPs at a concentration of 4000 μg/ml caused no significant threshold shifts upon click stimuli and tone burst stimuli at a frequency of 2 kHz, but significant threshold shifts from 5 dB to 33 dB at frequencies of 4 kHz through 32 kHz were observed (p < 0.01, independent sample t-test). No significant threshold shift was detected in rats exposed to AgNPs at a concentration of 200 μg/ml. There was a significant positive linear correlation between the threshold shift and frequency in rats exposed to AgNPs at a concentration of 4000 μg/ml at the observation time points of 2 d, 4 d, and 7 d (p < 0.01, 2-tailed Pearson correlation).

### Cell death in the rat inner ear after AgNP exposure

**Figure 5:**
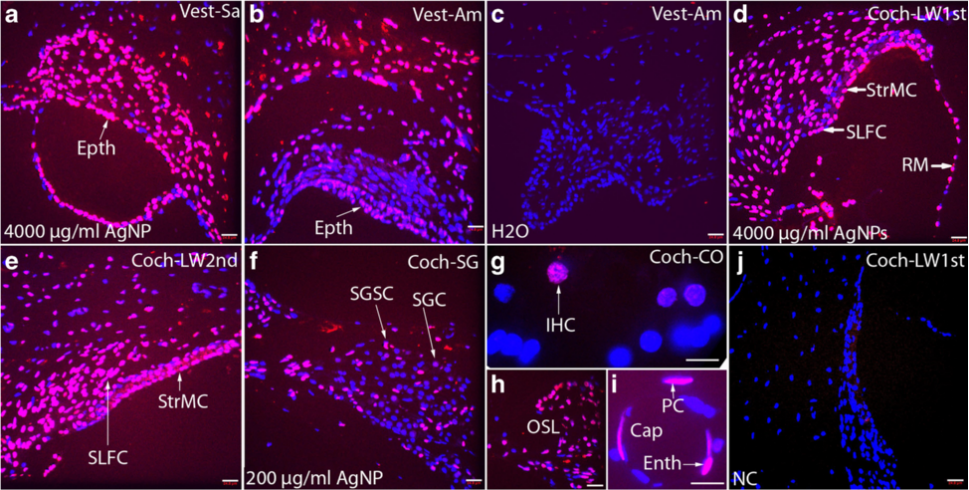
**DNA fragmentation in the inner ear cells at 7 d post-exposure to the AgNPs.** 4000 μg/ml AgNP caused DNA fragmentation in the cells in the vestibule **(a, b)**, stria vascularis **(d-f)**, Reissner’s membrane (RM) **(d, f)**, and spiral ligament (SL in **d**-**f**). In the lateral wall of second higher turn (Coch-LW2ndH), limited marginal cells (StrMC) in the stria vascularis and sparse fibrocytes in the spiral ligament (SL) showed DNA fragmentation **(f)**. 200 μg/ml AgNP caused DNA fragmentation in cells in osseous spiral lamina (OSL in **h**), cells in the cochlear capillary (Cap in I), and hair cells of the cochlear Corti’s organ (Coch-CO)(G). Water did not cause significant DNA fragmentation in the vestibule **(c)**. No staining was demonstrated in the negative control by omitting the labeling solution. Percentages of positive staining were 74%, 66%, 0%, 53%, and 59% in **a**, **b**, **c**, **d**, and E respectively. Coch-LW1stL: lateral wall of the cochlear lower basal turn; Coch-LW2ndL: lateral wall of the cochlear lower second turn; Coch-LW2ndH: lateral wall of the cochlear higher second turn; Enth: endothelial cells; Epth: epithelial cells; PC: pericytes of the Cap; Vest-Am: ampulla in the vestibule; Vest-Sa: saccule in the vestibule. Scale bars = 24.8 μm in **a**-**f**, **h**, **j**, and 10 μm in **g** and **i**.

Inner ear cell death (apoptosis) was detected in the inner ear exposed to AgNPs at concentrations of both 200 μg/ml and 4000 μg/ml, as indicated by DNA fragmentation. In general, greater cell death occurred in the vestibule than in the cochlea. This was more obvious at the lower concentration of AgNPs. In the cochlea, the strial marginal cells were among the most sensitive cells to develop apoptosis after AgNP exposure, and were followed by the osteocytes in the cochlear shell, the osseous spiral limbus, epithelial cells of the Reissner’s membrane, the spiral ligament fibrocytes, the spiral ganglion satellite cells, endothelial cells and pericytes of the cochlea capillaries, and cochlear hair cells. At 4000 μg/ml of AgNPs, there was universal cell death in the vestibule end organ and cochlea, between the hook region and the second lower turn, as well as the Reissner’s membrane, part of the spiral ganglion, sparse stria vascularis, spiral ligament, capillary, and osseous spiral limbus from the second higher turn to the apex. At 200 μg/ml AgNPs, there was cell death in most the vestibular end organ cells, and cochlear cell populations, which was similar to that caused by 4000 μg/ml AgNPs in the higher turns (Figure 5).

## Discussion

MRI results showed that the biological barriers in the skin, mucosa, and inner ear of rats were opened by the AgNPs in a dose-dependent manner, which was supported by histology. Changes in the biological barrier function of the ear were reversible in rats exposed to AgNPs at tested concentrations. The biological barriers impede the passage of substances as nanoparticles into the tissue and protect the organ so that it can function properly. Hence, they constrain the bioavailability of AgNPs. As the direct contact site, the barrier in the middle ear mucosa is the most accessible structure observed in the MRI study. The middle ear mucosa is a direct continuation of the mucosa of the upper respiratory tract; data acquired in the middle ear mucosa is relevant to explaining the impact of AgNPs on the respiratory mucosa, including the nasal mucosa.

After passing through the barriers of the round and oval windows, AgNPs entered the inner ear and disrupted the blood-perilymph and blood-endolymph barriers, causing hearing loss. Greater cell death in the vestibule than that in the cochlea indicated that the oval window is more permeable to AgNPs than the round window which is in accordance with the phenomenon observed in gadolinium transport from the middle ear to the inner ear [30]. However, no balance problem was observed in the animals exposed to any of these concentrations of AgNPs (data will be reported separately). Although AgNPs may directly damage the sensorineural cells in the inner ear, complete recovery of hearing loss induced by 200 μg/ml AgNPs suggested that most of the inner and outer hair cells and spiral ganglion cells are preserved, and hearing loss is mainly due to destabilized ion homeostasis [18,31,32]. The blood-endolymph barrier was rather resistant to AgNPs, suggesting that the blood-endolymph barrier integrity is critical to maintaining hearing function.

The present study showed that individual cells *in vitro* are more sensitive to AgNPs than the inner ear cells in vivo. It is in accordance with a previous study showing that individual primary cochlear cells are more sensitive to the mitochondrial toxin, 3-nitropropionic acid (3NP), than the cells in the cochlea of living guinea pigs [33]. In the present study, AgNP caused BALB/c 3T3 cell death with IC_50_ values ranging from 2.2 to 3.0 μg/ml, as determined by measuring the NAD(P)H-dependent cellular oxidoreductase enzyme activity (WST-1), total cellular ATP, nuclear membrane permeability (propidium iodide), and NRU. The observed significant increase in mitochondrial activity of BALB/c 3T3 cells at AgNP concentration 2.1 μg/ml without alteration in ATP levels indicated that the net ATP level might be affected by several activities. We suspect that an augmented cellular activity accompany an increased mitochondrial activity and the cells consume more ATP giving rise to a stable cellular ATP level as presented in the data. This indicates that WST-1 assay is a more sensitive and reliable parameter of cellular viability than measuring the ATP level. Therefore, WST-1 assay was selected for inter-laboratory validation in nanotoxicology of European Commission FP7 large-scale integrating project NanoValid (internal data) [34]. The IC_50_ for AgNO_3_ was lower than that for AgNPs. It has been reported that both AgNPs and ionic Ag^+^ (using AgNO_3_ as material control) are toxic to the cells [29]. This is actually doubtful because no AgNO_3_ remains in either the animal or human body after administration because AgNO_3_ reacts with saline and is converted to AgCl and $\mathrm{N}{\mathrm{O}}_3^{-}$ in plasma (and medium in the cell culture). It is well known that $\mathrm{N}{\mathrm{O}}_3^{-}$ ion is extremely toxic to any cells. If Ag^+^ is released from the AgNPs, the major product is AgCl, which has low water solubility. In the present study AgCl was, indeed, insignificantly toxic to the BALB/c 3T3 cells tested up to the saturated concentration. To correlate with in vitro studies, 200 μg/ml AgNPs induced reversible changes in the biological barrier and auditory functions. The dose of 4000 μg/ml AgNPs caused hearing loss (with partial recovery). In general, the IC_50_ in the present in vitro tests are more than 1000 times more sensitive than the in vivo studies, which is most likely attributed to the protective function of the highly regulated double layer of the biological barriers (the oval and round windows, and the endolymph barrier in the inner ear) and the extracellular matrix.

The hearing measurement was in line with the cell death observed in histology. At a dose of 200 μg/ml, AgNPs caused a reversible hearing loss at 32 kHz, correlating with the lower basal turn of the cochlea. This is in accordance with the cell death map (Figure 5). A dose of 4000 μg/ml AgNPs caused irreversible hearing loss above 8 kHz, matching the broadly distributed cell death up to the second lower turn (Figure 5) [35]. However, only partial hearing loss occurred at the frequencies of 16 kHz and 32 kHz instead of total loss on day 7. Our explanation for this disparity is that spatial information on Corti’s organ dysfunction provided by tone burst ABR is much less accurate than the histology. It is known that the tone burst, which has relatively broad spectra, also elicits a response from a region of intact Corti’s organ that is distant from the nominal stimulus frequency and causes a false positive result [36]. Therefore, the 16 kHz and 32 kHz stimuli elicited nerve fibers that are responsible for higher tone hearing where the Corti’s organs did not show any impairment at the higher turns when the Corti’s organs at their specific frequency were damaged.

The vestibular cells demonstrated greater impairment than the cochlear cells after exposure to AgNPs at a low concentration, indicating that the passage of AgNPs into the vestibule through the oval window might be more efficient than the passage into the cochlea through the round window because AgNP induced cellular toxicity is concentration-dependent, as shown in the in vitro study. However, this hypothesis needs to be tested in future studies.

Our results suggest that the AgNPs impaired mitochondrial function through inhibition of the mitochondrial succinate-tetrazolium reductase activity (shown by WST-1) and ATP production. The notable ~50% increase in mitochondrial activity (as shown by WST-1) at 2.1 μg/ml prior to its ~50% reduction at 3.0 μg/ml indicated the onset of defense process upon AgNP stimuli, i.e., hormesis (other cytotoxicity assays did not show any increase at this concentration) and further supported the occurrence of oxidative stress within the mitochondria [37]. These results also support the previously reported findings that showed that mitochondrial dysfunction is involved in hearing loss by causing energy shortage. The sensitivity of different cell populations to mitochondrial toxins is determined by the cellular activity-related energy consumption [18,33]. The cells that are impaired in the blood-perilymph and blood-endolymph barriers after the delivery of AgNPs are among the sensitive populations. A comprehensive gene sequencing study in zebrafish embryos demonstrated that the most notably affected gene pathway by silver in the nano-, bulk-, and ionic forms is associated with oxidative phosphorylation and protein synthesis [38]. The mitochondria is the site of oxidative phosphorylation, and AgNPs disrupt mitochondrial membrane permeability as well as decrease the activity of the mitochondrial respiratory chain complexes I, II, III, and IV in cells from the brain, skeletal muscle, heart, and liver of rats [39,40]. Specifically, AgNPs induced apoptosis in NIH3T3 cells was reportedly associated with the generation of reactive oxygen species (ROS) and Jun amino-terminal kinases (JNK) activation, leading to the release of cytochrome C into the cytosol and translocation Bax to mitochondria [26]. Consequently, the cell death of BALB/c 3T3 cells induced by AgNPs in the present study was associated with the disrupted integrity of the nuclear membrane, which was visible by propidium iodide staining.

In conclusion, AgNPs caused significant, dose-dependent changes in the permeability of biological barriers in the middle ear mucosa, the skin of the external ear canal, and the inner ear. The functional change in the auditory system was in line with the histology results. In general, the BALB/c 3T3 cell line is more than 1000 times more sensitive than the in vivo studies. Impairment of the mitochondrial function was indicated to be the toxic mechanism caused by the AgNP exposure. These results suggest that the concentration of AgNPs should be tightly controlled in clinic application in treating otitis media. The rat ear model might be expended to other engineered nanomaterials in nanotoxicology study.

## Materials and methods

The polyvinylpyrrolidone stabilized AgNPs was supplied by Colorobbia (Firenze, Italy). The AgNPs were dispersed in deionized water (40 mg/ml) and scanning electron microscopy showed that the AgNPs are spheroids in morphology with a particle size of around 100 nm. Dynamic light scattering (DLS) showed a mean hydrodynamic size of 117 ± 24 nm and a mean zeta potential of −20 ± 9 mV. The selected AgNPs is stable in artificial perilymph which is the main solution in the inner ear that AgNPs would interact (data including the full characterization data will be published separately).

The murine fibroblast cell line BALB/c 3T3 clone 31 was purchased from ATCC (American Type Culture Collection, LGC Promochem AB, Boras, Sweden). Dulbecco’s Modification of Eagle’s Medium (DMEM), L-glutamine, and newborn calf serum (NBCS) were purchased from Gibco Invitrogen (Carlsbad, USA). Stability of the AgNPs in the DMEM is still under inverstigation between several laboratory in Europe within the European Union 7^th^ frame programme (EU FP7) large-scale integrating project NanoValid [34] (http://cordis.europa.eu/result/rcn/140307_en.html). An ATP Determination Kit was purchased from Life Technologies (California, USA). WST-1 and an In Situ Cell Death Detection Kit (TMR red) were purchased from Roche (Basel, Switzerland). Propidium iodide, paraformaldehyde (PFA), 4,6-diamidino-2-phenylindole (DAPI), and Fluoromoun™ were purchased from Sigma-Aldrich (St. Louis, USA).

Fourteen male Sprague Dawley rats for the MRI study, weighing between 287 g and 524 g, were maintained in the Biomedicum Helsinki, Laboratory Animal Centre, University of Helsinki. Fourteen male Sprague Dawley rats for ABR measurements weighing between 330 g and 410 g, were maintained in the Experimental Animal Unit, School of Medicine, University of Tampere, Finland (Table 2). All animal experiments were approved by the Ethical Committee of University of Tampere (permission: ESAVI/3033/04.10.03/2011). Animal care and experimental procedures were conducted in accordance with European legislation. Animals for the MRI study were anesthetized with isoflurane with a 5% isoflurane–oxygen mixture (flow-rate 1.0 L/min) for induction and 3% for maintenance via a facemask. The ABR measurements were performed under general anesthesia after the intraperitoneal injection of a mixture of 0.8 mg/kg of medetomidine hydrochloride (Domitor, Orion, Espoo, Finland) and 80 mg/kg of ketamine hydrochloride (Ketalar; Pfizer, Helsinki, Finland). During the experiments, Viscotears® (Novartis Healthcare A/S, Denmark) were used to protect the animals’ eyes.

**Table 2:** **Assignments of rats in MRI and ABR measurements post-intratympanic administration of AgNPs**

**Measures**	**Number of rats exposured to varied concentration of AgNPs for different time**									
	**4000 μg/ml**				200 μg/ml*				**2000 μg/ml**	
	**5 h**	**2 d**	**4 d**	**7 d**	**5 h**	**2 d**	**4 d**	**7 d**	**5 h**	**7 d**
MRI	2	-	-	2	7	-	-	-	5	5
ABR	-	7	7	7	-	7	7	7	-	-

*Three rats receiving 400 μg/ml AgNP injection were pooled in the 200 μg/ml group in MRI study. ABR: auditory brainstem response. -: no exposure.

BALB/c 3T3 cells were cultured in DMEM containing 4 mM L-glutamine and supplemented with 10% NBCS at 37°C with 5% CO_2;_ they were then seeded to 96-well plates at a density of ~3000 cells/well and allowed to form a 50-70% confluent monolayer after 24 ± 2 h. For the propidium iodide assays, 96-well black plates with clear bottoms were used. After replacing the medium with 90 μl DMEM containing 4 mM L-glutamine and 5% NBCS, 10 μl AgNP dilutions (prepared immediately prior to use in deionized water) were added to the wells to reach eight different AgNP and AgNO_3_ (final concentrations 0.67-10.0 μg/ml) and six different AgCl (final concentrations 0.0052-5.2 μg/ml) concentrations with 6 replicates. The plates were then incubated for 24 h. The exposure time was defined according the results showing that AgNPs remained in rat cochlear for at least 24 h after transtympanic injection (unpublished data acquired using micro computed tomography). The medium containing 10% deionized water was used as a vehicle control. Four different assays were used to study the viability of BALB/c 3T3 cells after AgNP exposure, which are NRU, WST-1, the total cellular ATP, and propidium iodide staining. Each assay was performed twice (Additional file 1: Support material 1).

Under general anesthesia, 40–50 μl of AgNPs at defined concentrations were injected into the left middle ear cavity through the tympanic membrane penetration under an operating microscope (Table 2). After injection, the animals were kept in the lateral position with the injected ear oriented upward for 15 min before further measurements.

A 4.7 T MR scanner with a bore diameter of 155 mm (PharmaScan, Bruker BioSpin, Germany) was used in the MR measurements for evaluation of the biological barrier function in the ear. The maximum gradient strength was 300 mT/m with an 80-μs rise time. A gadolinium-tetra-azacyclo-dodecane-tetra-acetic acid (Gd-DOTA, 500 mM, DOTAREM, Guerbet, Cedex, France) solution was injected into the tail vein at a dosage of 0.725 mM/kg 2 h before the MRI measurements. MRI scanning commenced at several time points after the transtympanic injection. The first MRI time of 5 h was determined by taking the penetration time of liposome nanoparticles from the middle ear to the inner ear as a reference [16]. The final imaging time of 7 d was selected according to the course of acute inflammation. For imaging protocols, refer to the Additional file 1: Support material 2.

BioSig32 (Tucker Davis Technologies, Florida, USA) was used for the ABR threshold recording in rats in a custom made, soundproof chamber. Both click and tone burst stimuli were used for the ABR measurements at a certain time point post-administration of AgNPs. The first ABR measurement was followed on 2 d post-administration of AgNPs allowing the animals to recover from the general anesthesia during the injection and ensure the injected solution to be entirely cleared from the middle ear cavity. The second follow-up time of 4 d post-injection was chosen because it is close to the peak time of mitochondrial toxin-induced cell death in the cochlea [33]. The third follow-up time of 7 d is the period of acute inflammation. For details on the ABR recording, refer to the Additional file 1: Support material 3.

For nuclear DNA fragmentation analysis in rat cochlea, animals were perfused with 4% PFA in 0.1 M PBS (pH 7.4) following cardiac perfusion and the removal of the blood with 50 mL physiological saline containing 0.3 mL heparin (100 IE). The bullae were collected and fixed with 4% PFA overnight and processed for nuclear DNA fragmentation investigation using terminal transferase (TdT) to label the free 3’OH breaks in the DNA strands of apoptotic cells with TMR-dUTP. For details on the analysis, refer to the Additional file 1: Support material 4.

## Additional file

Additional file 1:
**Support materials 1-4.**

## Abbreviations

ABR: Auditory brainstem response
AgNPs: Silver nanoparticles
DAPI: 4,6-diamidino-2-phenylindole
DMEM: Dulbecco’s Modification of Eagle’s Medium
Gd-DOTA: Gadolinium-tetra-azacyclo-dodecane-tetra-acetic acid
JNK: Jun amino-terminal kinases
MRI: Magnetic resonance imaging
NBCS: Newborn calf serum
NRU: Neutral red uptake
OECD: Organization for Economic Cooperation and Development
PFA: Paraformaldehyde
ROS: Reactive oxygen species
TdT: Terminal transferase
