# Supplementary material for: Toxicity of silver nanoparticle in rat ear and BALB/c 3T3 cell line
Source: J Nanobiotechnology. 2014 Dec 3;12:52. doi: 10.1186/s12951-014-0052-6 (PMC4272548; doi:10.1186/s12951-014-0052-6)
Supplement: Additional file 1: — Support materials 1-4. [file 12951_2014_52_MOESM1_ESM.docx]

**Additional file** **1**

*Support material 1*

In NRU assay, exposure medium was removed and the plates were washed with D-PBS (250 µl/well). NR-solution (25 µl/ml neutral red prepared in DMEM supplemented with 5 % NBCS, 100 IU/ml penicillin and 100 µg/ml streptomycin) was added to all wells. The plate was incubated at 37°C, 5% CO2 for 3.0±0.1 hrs. NR-solution was removed, and plates were washed with D-PBS (250 µl/well). 100 µl of NR desorption solution (40 parts water, 50 parts ethanol, 10 part acetic acid) was added to all wells. Plate was shaken for 30 minutes protected from light, kept still for about 5 minutes, and absorbance was read at 540±10 nm.

In WST-1 assay, 10 µl of WST-1 reagent was added into each well in the end of exposure. The plate was incubated at 37°C with 5% CO2 for 180 ± 10 min. After incubation, the plate was shaken for 1 min and read at 450±10 nm (OD450).

In ATP measurement, 5 µl of 10% TCA was added to each well after the exposure. The plates were agitated and frozen at -80°C overnight before they were thawed and agitated. The standard reaction solution of the ATP Determination Kit was pipetted into each well of a new 96-well plate at 100 µl/well. The reaction was initiated by adding 10 µl of sample to each well. Then, luminescence was measured.

In propidium Iodide assay, 25 µl of 100 µg/ml propidium iodide solution was added to each well (final concentration of 10 µg/ml) in the end of exposure. After incubation in the dark at room temperature for ~10 min, the fluorescence was measured (excitation 530 nm, emission 620 nm). The plates were then frozen at ~-20°C overnight. After thawing, the fluorescence was measured as above. The difference between these two measurements represented the number of living cells.

The absorbance, luminescence and fluorescence were measured using a Varioskan Flash Multimode Reader (Thermo Scientific). The cell viability was expressed as a percentage of the viability of treated cells relative to that of the untreated (vehicle) control wells. Dose-response curves were drawn, and the IC50 values calculated using SigmaPlot®.

*Support material 2*

The body temperature of the rats was maintained by circulating warm water, and respiration was recorded with the Physio Tool-1.0.b.2 program (Bruker, Germany). Rats were placed in the magnet with their ears positioned at the isocenter. T2-weighted 2D images were acquired using rapid acquisition with relaxation enhancement (RARE) sequences (TR/TEeff 2500/40 ms, RARE factor 8, matrix size 256x256, slice thickness [ST] 0.8 mm, field of view [FOV] 5.0x5.0 cm^2^, resolution 0.195x0.195 mm^2^, number of averages [NEX] 3) to set up the geometry. T1-weighted 2D images were acquired with a RARE sequence (TR/TEeff 500/10 ms, RARE factor 4, matrix size 256x192, slice thickness 0.5 mm, FOV 2.5x2.5 cm^2^, resolution 0.098x0.13 mm^2^, NEX 33). A 2D fluid-attenuated inversion-recovery (FLAIR) sequence (TR/TE_eff_ 8000/40 ms, inversion time 1800 ms, RARE factor 16, matrix size 256 × 192, slice thickness 0.5 mm, FOV 3.0 × 3.0 cm^2^, resolution 0.117 × 0.156 mm^2^, NEX 7) was used to confirm the gadolinium signal. ParaVision PV 4.0 (Bruker, Germany) software was used for post-processing and quantification of MR images.

*Support material 3*

For the click ABR measurements, a click with a duration of 50 µs and a repetition rate of 21.1/s that was generated in a free field speaker (TDH, Telephonics, New York USA) was delivered into the external auditory canal using a pipette tip that was connected to a silica tube and plastic ear scope as well as mounted to the speaker as stimulation. Responses from 500 sweeps were averaged with a gain of 20 at each intensity level, using a filter of 0.1–3 kHz. In the frequency specific ABR measurements, the tone burst of decreasing intensity (10 dB decrements) with a duration of 47.619 ms and a repetition rate of 21/s was generated by a FF1 speaker (Tucker Davis Technologies, Florida, USA), which was placed 5 cm away from the opening of the external auditory canal, while the contralateral external auditory canal was plugged using a rubber insert. The responses from 1000 sweeps were averaged with a gain of 20 at each intensity level, using a filter of 0.3–3 kHz. The thresholds were identified as the minimum visible, repeatable response.

*Support material 4*

After washing with 0.01 M PBS, the specimens were decalcified with 10% EDTA at room temperature for 4 weeks, embedded with paraffin, and sectioned at 3 µm thickness. After dewaxing and rehydrating, the slides were incubated with proteinase K (Fermentas, USA, 20 μg/ml in 10 mM Tris/HCL, pH 7.4) for 30 min at 37°C. The slides were washed with PBS and then incubated with terminal deoxynucleotidyl transferase-mediated dUTP-biotin nick end labeling reaction mixture for 60 min at 37°C in the dark, followed by incubation with DAPI (10 µg/ml) for 10 minutes. The slides were again washed with PBS and then mounted with Fluoromoun^TM^. For positive controls, the slides were incubated with recombinant DNase I (Thermo scientific, USA, 100 U/ml in 10 mM Tris/HCL, pH 7.5, 2.5 mM MgCl2, 0.1 mM CaCl2) for 10 min at 37°C prior to the labeling procedures. For negative controls, the labeling solution was omitted from the reaction mixture. The reaction was visualized by confocal microscope utilizing a Nikon inverted microscope (ECLIPSE Ti) combined with an Andor confocal system that installed with Andor iQ 2.8 software (Andor Technology, Belfast, UK). The excitation lasers were 405 nm (blue excitation) and 568 nm (red excitation) from an Andor laser combiner system of the confocal system, and the corresponding emission filters were 450-465 nm (DAPI) and 607/45 nm (TMR).
